# Effect of Urgent ERCP on Clinical Outcomes in Acute Cholangitis With Concurrent Acute Gallstone Pancreatitis: A Propensity Score Matching Analysis

**DOI:** 10.1002/jhbp.12164

**Published:** 2025-06-05

**Authors:** Mustafa Comoglu, Fatih Acehan, Enes Seyda Sahiner, Huseyin Camli, Zeki Mesut Yalin Kilic, Bulent Odemis, Ihsan Ates

**Affiliations:** ^1^ Department of Internal Medicine Ankara Bilkent City Hospital Ankara Turkiye; ^2^ Department of Gastroenterology Ankara Bilkent City Hospital Ankara Turkiye

**Keywords:** acute cholangitis, acute gallstone pancreatitis, choledocholithiasis, propensity score, urgent ERCP

## Abstract

**Background/Purpose:**

Current guidelines do not provide specific recommendations regarding the timing of endoscopic retrograde cholangiopancreatography (ERCP) in patients with acute cholangitis (AC) concurrent with acute gallstone pancreatitis (AGP). This study evaluated the impact of ERCP timing on clinical outcomes.

**Methods:**

A total of 144 patients diagnosed with AC concurrent with AGP between March 2019 and February 2024 were included in the study. Patients were classified into two groups: urgent ERCP group (ERCP ≤ 24 h) and non‐urgent ERCP group (ERCP 24–72 h). Clinical outcomes were compared using propensity score matching (PSM) analysis.

**Results:**

After PSM, two well‐balanced groups of 55 patients were created. The median ERCP time was 18 (13–21) hours in the urgent group and 41 (36–54) hours in the non‐urgent group. There was no significant difference in composite outcomes, including in‐hospital mortality, prolonged hospital stay, severe pancreatitis, or late localized/systemic complications of pancreatitis [11 (20%) vs. 16 (29.1%); *p* = 0.268]. Additionally, no significant difference was observed between the groups regarding prolonged hospital stay (*p* = 0.506), ICU admission (*p* = 0.680), or in‐hospital mortality (*p* = 0.161).

**Conclusions:**

Urgent ERCP within 24 h does not significantly improve clinical outcomes compared to ERCP performed within 24–72 h in patients with AC and AGP.

## Graphical abstract

Comoglu and colleagues investigated the impact of urgent endoscopic retrograde cholangiopancreatography in patients with acute cholangitis and concurrent acute gallstone pancreatitis, a population lacking specific guideline recommendations. Although intervention within 24 h showed a favorable trend in severe cases, it did not significantly improve outcomes compared with 24–72 h.

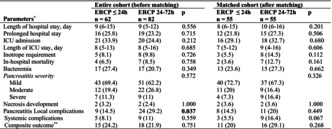

## 1 Introduction

Acute cholangitis (AC) is a potentially life‐threatening condition that requires prompt diagnosis and treatment to prevent serious complications, including sepsis and multi‐organ failure [1]. Concomitant acute gallstone pancreatitis (AGP) in patients with AC presents a unique clinical challenge, as these patients often exhibit a more severe disease course and are at higher risk for adverse outcomes [2]. The initial management of AC involves hemodynamic stabilization, broad‐spectrum antibiotics, and timely biliary drainage [3]. Endoscopic retrograde cholangiopancreatography (ERCP) is a cornerstone of biliary drainage, often performed urgently in severe cases [4].

The most frequently referenced guideline for determining the timing of ERCP in current clinical practice is the Tokyo Guidelines 2018 (TG18) [5]. According to TG18 and other relevant guidelines, ERCP is recommended within 24 h for severe AC, within 48–72 h for moderate cases, and may be scheduled electively for mild cases [5, 6]. However, the role of ERCP in AGP remains an area of ongoing research, with conflicting evidence regarding its impact on patient outcomes. While some studies suggest that urgent ERCP improves prognosis in AGP, others report no significant benefit [7, 8, 9]. Current AGP guidelines also lack clear recommendations on this issue [10, 11]. Additionally, spontaneous biliary drainage has been reported in 20%–73% of patients with choledocholithiasis [12, 13]. Given that gallstones responsible for AGP are typically located in the distal biliary tract, the likelihood of spontaneous passage into the duodenum may be higher in this patient population [14]. These factors further complicate the decision‐making process regarding the optimal timing of ERCP in patients with concurrent AC and AGP.

While previous studies suggest that urgent ERCP (≤ 24 h) improves outcomes in AC, its impact in patients with coexisting AGP remains unclear due to limited and inconclusive data. In this study, we aimed to assess the effect of urgent ERCP on clinical outcomes in patients with AC and concomitant AGP. By comparing outcomes between those who underwent urgent ERCP and those who received the procedure within 24–72 h, we sought to determine whether early intervention confers a prognostic advantage in this unique patient population.

## 2 Materials and Methods

### 2.1 Study Design and Clinical Outcomes

**FIGURE 1:**
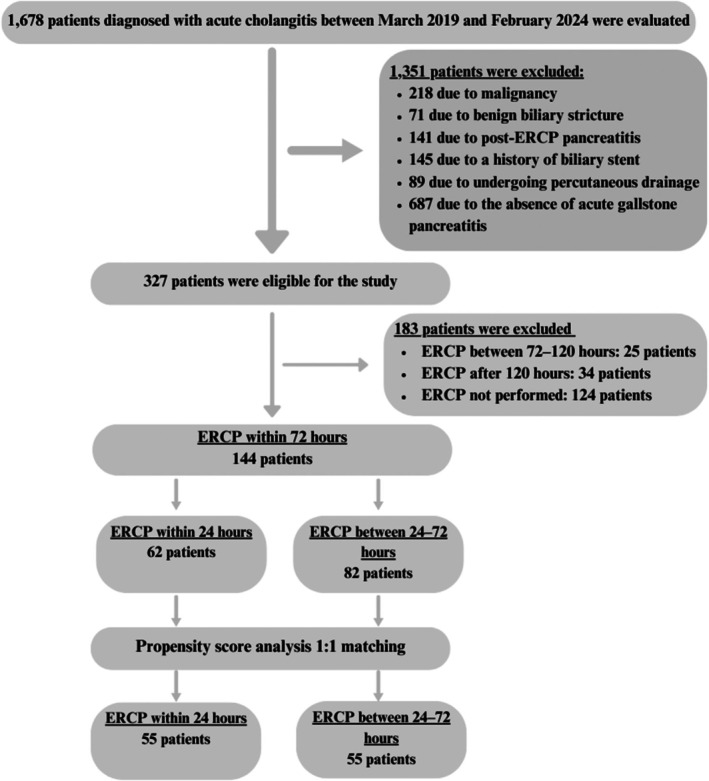
Flow chart of the study.

This retrospective cohort study was conducted in the Internal Medicine Department of Ankara Bilkent City Hospital, including patients diagnosed with AC and concomitant AGP between March 2019 and February 2024. Ethical approval for the study was obtained from the Ankara Bilkent City Hospital Ethics Committee (decision no. 2‐24‐442). The inclusion criteria were being over 18 years of age and having a diagnosis of AC and AGP due to gallstones or biliary sludge. The exclusion criteria were the presence of malignancy, acute pancreatitis or cholangitis etiologies other than gallstones, chronic pancreatitis, post‐ERCP acute pancreatitis, undergoing percutaneous transhepatic cholangiography, and a history of biliary stents or benign biliary stricture. Additionally, patients who did not undergo ERCP within the first 72 h were excluded. A total of 144 patients meeting these criteria were included in the study (Figure 1).

The time from the patients' admission to ERCP was recorded. Based on this, the patients were divided into two groups: those who underwent ERCP within 24 h (urgent ERCP group) and those who underwent ERCP between 24 and 72 h (non‐urgent ERCP group). These two groups were compared in terms of clinical outcomes. The primary composite endpoint was defined as in‐hospital mortality, prolonged hospital stay, severe pancreatitis, or the development of late localized or systemic complications of pancreatitis. Secondary endpoints included intensive care unit (ICU) admission, length of ICU stay, in‐hospital mortality, development of necrosis, prolonged hospital stay, occurrence of systemic or local complications of pancreatitis, and inotrope requirement.

### 2.2 Definitions

The diagnosis and severity grading of AC were based on the TG18 [5]. To confirm a diagnosis of AC, patients had to meet all three of the following criteria: (1) evidence of systemic inflammation (body temperature > 38°C, white blood cell [WBC] count > 10 × 10^9^/L, or C‐reactive protein [CRP] level > 10 mg/L); (2) evidence of cholestasis, including jaundice (total bilirubin ≥ 2 mg/dL) and abnormal liver enzymes (≥ 1.5 times the upper limit of normal); and (3) imaging findings indicating biliary dilation or an underlying etiology (e.g., stones) [5]. Biliary dilatation was defined as a common bile duct diameter of > 8 mm in patients aged ≤ 75 years and > 10 mm in patients aged > 75 years or those with a history of cholecystectomy. According to the TG18, the severity of AC is classified into three grades. Severe AC (Grade III) is defined by the presence of at least one organ/system dysfunction, including cardiovascular, neurological, respiratory, renal, hepatic, or hematological failure. Moderate AC (Grade II) is diagnosed when two or more of the following criteria are present: abnormal WBC count (< 4 × 10^9^/L or > 12 × 10^9^/L), fever ≥ 39°C, age ≥ 75 years, total bilirubin ≥ 5 mg/dL, or hypoalbuminemia (< 70% of the lower normal limit). Mild AC (Grade I) refers to cases that do not meet the criteria for severe or moderate AC [5].

All definitions related to AGP were based on the 2012 Revised Atlanta Classification [15]. The diagnosis of AGP was established by the presence of at least two of the following three criteria: (1) abdominal pain characteristic of AGP, (2) serum amylase and/or lipase levels exceeding three times the upper limit of the normal reference range, and (3) radiological evidence of pancreatic and/or peripancreatic inflammation consistent with AGP. Patients with imaging findings of biliary sludge, gallstones, or a dilated common bile duct were classified as having AGP. The severity of pancreatitis was classified according to the Revised Atlanta Classification into three categories: mild cases without complications or organ failure, moderately severe cases with complications and/or organ failure lasting less than 48 h, and severe cases with organ failure persisting for more than 48 h. Organ failure was defined as a score of 2 or higher according to the modified Marshall scoring system [16]. Local complications were classified as acute peripancreatic fluid collection, pseudocysts, walled‐off necrosis, and acute necrotic collection. Late localized complications were defined as the development of pseudocysts and necrosis. The exacerbation of an underlying comorbid disease was defined as a systemic complication, while splanchnic venous thrombosis and pseudoaneurysms were categorized as vascular complications [15].

General demographic characteristics, comorbidities, vital signs, presenting complaints, laboratory findings at admission, Charlson comorbidity index (CCI) values [17], length of hospital stay, ICU admission, presence of bacteremia, in‐hospital mortality, and prolonged hospital stay were obtained from electronic medical records. Patients presenting from Friday evening until Sunday night were defined as weekend admissions. Prolonged hospital stay was defined as a length of stay > 14 days before matching and > 15 days after matching, corresponding to the 75th percentile in each group.

### 2.3 Patient Management

All patients diagnosed with AC received appropriate intravenous fluid replacement and antibiotic therapy immediately after diagnosis. Blood cultures were obtained before initiating antibiotic therapy, and the treatment was adjusted based on culture results by the infectious diseases team. The institutional criteria guiding the timing of ERCP at our hospital included the initial Tokyo severity grading at presentation, the degree of cholestasis and elevation in acute‐phase reactants, lack of improvement in cholestatic enzymes (particularly total bilirubin) during follow‐up, and evidence of hemodynamic deterioration. During clinical follow‐up, magnetic resonance cholangiopancreatography (MRCP) was performed in patients who showed regression in cholestasis enzymes and acute‐phase reactants, as well as improvement in clinical status after antibiotic therapy and intravenous fluid treatment, to determine whether spontaneous biliary drainage had occurred.

### 2.4 Propensity Score Matching and Statistical Analysis

The covariates included in the PSM analysis consisted of demographic characteristics (age and CCI score), factors associated with the severity of AC and AGP upon admission (Tokyo severity grading, bedside index of severity in acute pancreatitis [BISAP] score, and CRP), and weekend admission status. Age and CCI are not only well‐established prognostic factors in patients with biliary diseases but may also act as potential confounders, as clinicians might be more inclined to delay invasive procedures such as ERCP in elderly patients or those with a higher comorbidity burden. The Tokyo Guidelines severity grading [5] and the BISAP [18] score are widely accepted tools for assessing the severity of AC and AGP, respectively, and are strongly associated with prognosis. CRP was included as an objective inflammatory marker reflecting disease activity at admission. Additionally, weekend admission has been reported to affect the timing and outcomes of emergent gastrointestinal procedures and was therefore considered a relevant covariate [19].

All patients were assigned a propensity score representing the probability of group assignment given a set of observed covariates. Following the assignment of the propensity scores, a dataset was generated by matching patients using a simple 1:1 nearest‐neighbor method without replacement and a caliper width equal to 0.2. Examining standardized mean differences, an imbalance was defined as an absolute value greater than 0.20 (small effect size).

Statistical assessments were performed using the Statistical Package for the Social Sciences (SPSS) for Windows, (version 22) and the R program (version 2.15.3 for Windows). To incorporate these programs and perform PSM analysis, developer‐based software providing a custom dialog in the SPSS menu was used [20]. The Student's *t*‐test and Mann–Whitney *U* test were used to compare two continuous variables with and without a normal distribution, whereas the Pearson chi‐square and Fisher exact tests were utilized for the comparison of categorical variables. In all analyses, *p* < 0.05 was considered statistically significant.

## 3 Results

### 3.1 Balance of Covariate Distribution Between Matched Groups

We assessed the relative multivariate imbalance L1 measure, which was greater in the unmatched sample (0.745) compared to the matched sample (0.727). This indicates that our matching process achieved balance. In addition, the standardized mean differences for each of the six analyzed covariates were < 0.2, confirming the state of balance (Table 1).

**TABLE 1:** Comparison of general demographic and clinical characteristics.

Parameters^a^	Entire cohort (before matching)				Matched cohort (after matching)			
	ERCP ≤ 24 h *n* = 62	ERCP 24–72 h *n* = 82	*p*	SMD	ERCP ≤ 24 h *n* = 55	ERCP 24–72 h *n* = 55	*p*	SMD
Matched variables								
Age, years	68 ± 16	65 ± 17	0.487	−0.205	68 ± 17	66 ± 17	0.598	−0.101
CCI score	1 (0–3)	0 (0–1)	**0.032**	−0.348	1 (0–2)	0 (0–2)	0.588	0.000
Severe cholangitis	22 (35.5)	17 (20.7)	**0.049**	−0.362	16 (29.1)	17 (30.9)	0.835	0.045
BISAP score > 2	15 (24.2)	13 (15.85)	0.211	−0.227	13 (23.6)	12 (21.8)	0.820	−0.049
Weekend admission	11 (17.7)	26 (31.7)	0.058	0.298	10 (18.2)	11 (20)	0.808	0.039
C‐reactive protein, mg/L	100 (33–180)	47 (17–130)	**0.011**	−0.528	83 (27–150)	54 (24–152)	0.371	−0.195
Female gender	29 (46.8)	39 (47.6)	0.925		27 (49.1)	28 (50.9)	0.849	
Comorbidities								
Diabetes mellitus	20 (32.2)	23 (28)	0.585		14 (25.5)	15 (27.3)	0.829	
Hypertension	34 (54.8)	31 (37.8)	**0.042**		28 (50.9)	19 (34.5)	0.083	
Cardiovascular disease	18 (29)	15 (18.3)	0.129		15 (27.3)	12 (21.8)	0.618	
Cerebrovascular disease	3 (5)	3 (3.6)	1.000		1 (1.8)	3 (5.5)	0.506	
Abdominal pain	61 (98.4)	82 (100)	0.431		54 (98.2)	55 (100)	1.000	
Jaundice	28 (45.2)	37 (45.1)	0.996		22 (40)	26 (47.3)	0.442	
Vital signs								
Mean arterial pressure	86 (73–93)	89 (79–93)	0.403		86 (73–93)	88 (76–93)	0.683	
Heart rate per minute	86 (78–100)	82 (77–98)	0.423		86 (80–98)	87 (78–107)	0.789	
Oxygen saturation, %	95 (92–96)	95 (93–97)	0.118		94 (92–96)	95 (93–97)	0.197	
Glasgow Coma score < 15	6 (9.7)	4 (4.9)	0.328		5 (9.1)	4 (7.3)	1.000	
Recurrent pancreatitis	3 (4.8)	10 (12.2)	0.127		3 (5.5)	6 (10.9)	0.489	
Concomitant cholecystitis	13 (21)	14 (17.1)	0.553		11 (20)	9 (16.4)	0.621	
History of cholecystectomy	9 (14.5)	18 (22)	0.258		9 (16.4)	11 (20)	0.621	
Imaging finding on admission			0.123				0.243	
Biliary dilatation	23 (37.1)	41 (50)			19 (34.5)	25 (45.5)		
CBD stones or sludge	39 (62.9)	41 (50)			36 (65.5)	30 (54.5)		
Gallbladder stones or sludge	43 (69.4)	57 (69.5)	0.984		37 (67.3)	39 (70.9)	0.680	

*Note:* Significant *p* values appear in bold.

Abbreviations: BISAP, bedside index of severity in acute pancreatitis; CBD, common bile duct; CCI, Charlson comorbidity index; ERCP, endoscopic retrograde cholangiopancreatography; SMD, standardized mean difference.

^a^ Categorical variables are presented as *n* (%), non‐normally distributed numerical variables as median (first quartile, third quartile), and normally distributed numerical variables as mean ± standard deviation.

### 3.2 Comparative Baseline Characteristics Before and After Matching

Among the 144 patients included in the study, 62 were in the urgent ERCP group, while 82 were in the non‐urgent ERCP group. There were no significant differences between the groups in terms of age or sex (*p* = 0.487 and *p* = 0.925, respectively). The CCI score was higher in the urgent ERCP group (1 [0–3] vs. 0 [0–1], *p* = 0.032). Hypertension was more common in the urgent ERCP group (54.8% vs. 37.8%, *p* = 0.042). Radiological findings, including biliary dilation, stones, or sludge, were assessed at admission, with no significant differences between the groups (*p* = 0.123).

Using PSM analysis at a 1:1 ratio, 55 patients were assigned to the urgent ERCP group and 55 to the non‐urgent ERCP group. After matching, there were no significant differences between the groups in terms of CCI score, comorbidities, and other baseline characteristics (Table 1).

### 3.3 Comparison of Disease Severity and Laboratory Parameters

While cholangitis severity was higher in the urgent ERCP group before matching (*p* = 0.033), there were no differences between the groups after matching (*p* = 0.613). Before matching, Ranson, BISAP, and APACHE II scores were significantly higher in the urgent ERCP group (*p* = 0.045, *p* = 0.023, and *p* = 0.004, respectively); however, no significant differences were observed post‐matching. Procalcitonin and CRP levels were significantly higher in the urgent ERCP group before matching (*p* < 0.001 and *p* = 0.011, respectively), but no significant differences were found post‐matching (*p* = 0.102 and *p* = 0.371, respectively). Creatinine levels were higher in the urgent ERCP group both before and after matching (*p* < 0.001 and *p* = 0.005, respectively). The comparison of other disease severity and laboratory parameters is presented in Table 2.

**TABLE 2:** Comparison of classifications of disease severity and laboratory parameters.

Parameters^a^	Entire cohort (before matching)			Matched cohort (after matching)		
	ERCP ≤ 24 h *n* = 62	ERCP 24–72 h *n* = 82	*p*	ERCP ≤ 24 h *n* = 55	ERCP 24–72 h *n* = 55	*p*
Cholangitis severity			**0.033**			0.324
Grade I (mild)	17 (27.4)	39 (47.6)		17 (30.9)	23 (41.8)	
Grade II (moderate)	23 (37.1)	26 (31.7)		22 (40)	15 (27.3)	
Grade III (severe)	22 (35.5)	17 (20.7)		16 (29.1)	17 (30.9)	
qSOFA score ≥ 2	6 (9.7)	4 (4.9)	0.328	5 (9.1)	4 (7.3)	1.000
SIRS score ≥ 2	21 (33.9)	20 (24.4)	0.212	19 (34.5)	19 (34.5)	1.000
Scoring systems						
Ranson	3 (2–4)	3 (1–4)	**0.045**	3 (2–4)	3 (2–4)	0.320
BISAP	2 (1–2)	1 (0–2)	**0.023**	2 (1–2)	1 (0–2)	0.398
APACHE II	8 (5–11)	6 (3–8)	**0.004**	8 (5–10)	6 (3–8)	0.243
Glasgow‐Imrie	2 (1–2)	1 (1–2)	0.192	2 (1–2)	1 (1–2)	0.485
Organ failure at admission	11 (17.7)	6 (7.3)	0.055	7 (12.7)	5 (9.1)	0.541
CRP > 150 mg/dL, first 48 h	32 (51.6)	28 (34.1)	**0.035**	27 (49.1)	25 (45.5)	0.702
Pleural effusion, first 48 h	5 (8.1)	7 (8.5)	0.919	5 (9.1)	4 (7.3)	1.000
Laboratory parameters						
White blood cell count, 10^9^/L	12.3 (9.2–16.2)	11.9 (9.1–15.1)	0.280	12.2 (9.1–17.7)	12 (9–15)	0.371
Hemoglobin, g/dL	13.5 ± 1.9	13.8 ± 2	0.309	13.5 ± 1.8	13.6 ± 2	0.917
Platelet count, 10^9^/L	212 (171–286)	236 (190–282)	0.254	222 (172–288)	230 (186–293)	0.660
Urea, mg/dL	47 (36–75)	39 (28–51)	**0.001**	47 (34–72)	39 (28–58)	0.062
Creatinine, mg/dL	1.2 (0.9–1.6)	0.9 (0.7–1.1)	**< 0.001**	1.2 (0.9–1.5)	0.9 (0.7–1.2)	**0.005**
Albumin, g/dL	3.8 (3.5–4.2)	4 (3.6–4.4)	0.099	3.8 (3.5–4.2)	4 (3.6–4.4)	0.285
AST, U/L	181 (109–298)	173 (95–298)	0.641	180 (110–305)	177 (94–355)	0.652
ALT, U/L	190 (140–341)	215 (119–352)	0.536	197 (140–341)	210 (112–422)	0.795
Amylase, U/L	855 (536–1456)	921 (377–1843)	0.921	977 (592–1635)	880 (458–1843)	0.699
Lipase, U/L	1491 (796–2234)	1217 (540–2867)	0.705	1500 (802–2388)	1270 (540–2911)	0.649
Total bilirubin, mg/dL	5.2 (3.6–9.1)	5.4 (3–7)	0.475	4.4 (3.1–7.6)	5.3 (3–7)	0.738
Procalcitonin, μg/L	5.82 (0.4–21.9)	0.55 (0.2–4.5)	**< 0.001**	5.9 (0.32–23)	0.86 (0.2–14.4)	0.102
C‐reactive protein, mg/L	100 (33–180)	47 (17–130)	**0.011**	83 (27–150)	54 (24–152)	0.371

*Note:* Significant *p* values appear in bold.

Abbreviations: ALT, alanine aminotransferase; AST, aspartate aminotransferase; ERCP, endoscopic retrograde cholangiopancreatography; qSOFA, quick sequential organ failure assessment; SIRS, systemic inflammatory response syndrome.

^a^ Categorical variables are presented as *n* (%), non‐normally distributed numerical variables as median (first quartile, third quartile), and normally distributed numerical variables as mean ± standard deviation.

### 3.4 Comparative Analysis of ERCP Techniques

Following PSM, the median time from admission to ERCP was 18 [12, 13, 14, 15, 16, 17, 18, 19, 20, 21] hours in the urgent group and 39 (35–48) hours in the non‐urgent group. Cannulation techniques were comparable between the two groups (*p* = 0.664). Similarly, there was no significant difference in the rate of incomplete procedures [11 (20%) vs. 9 (16.4%), *p* = 0.621] or in ERCP‐related complications (*p* = 1.000) (Table 3).

**TABLE 3:** Comparative analysis of ERCP techniques and findings between groups.

Parameters^a^	Entire cohort (before matching)			Matched cohort (after matching)		
	ERCP ≤ 24 h *n* = 62	ERCP 24–72 h *n* = 82	*p*	ERCP ≤ 24 h *n* = 55	ERCP 24–72 h *n* = 55	*p*
Time from admission to ERCP, hours	18 (13–21)	41 (36–54)	**< 0.001**	18 (12–21)	39 (35–48)	**< 0.001**
Choledochal duct cannulation			0.473			0.664
Normal cannulation	46 (74.2)	65 (79.3)		42 (76.4)	44 (80)	
Pre‐cut incision	3 (4.8)	5 (6.1)		2 (3.6)	5 (9.1)	
Double guidewire technique	6 (9.7)	7 (8.5)		6 (10.9)	4 (7.3)	
Fistulotomy	7 (11.3)	5 (6.1)		5 (9.1)	2 (3.6)	
Choledochal stent placement	54 (87.1)	66 (80.5)	0.292	48 (87.3)	45 (81.8)	0.429
Pancreatic duct cannulation	7 (11.3)	13 (15.9)	0.433	7 (12.7)	8 (14.5)	0.781
Pancreatic duct stent placement	7 (11.3)	11 (13.4)	0.703	7 (12.7)	7 (12.7)	1.000
Endoscopic sphincterotomy	48 (77.4)	70 (85.4)	0.220	44 (80)	46 (83.6)	0.621
ERCP finding			0.284			0.613
Presence of stones	49 (79)	55 (67.1)		42 (76.4)	39 (70.9)	
Presence of sludge/debris	10 (16.1)	21 (25.6)		10 (18.2)	14 (25.5)	
Clear biliary ducts	3 (4.8)	6 (7.3)		3 (5.5)	2 (3.6)	
Stone or sludge extraction	54 (87.1)	66 (80.5)	0.292	47 (85.5)	47 (85.5)	1.000
Incomplete ERCP procedure	12 (19.4)	11 (13.4)	0.335	11 (20)	9 (16.4)	0.621
Balloon dilation during ERCP	3 (4.8)	0	0.078	3 (5.5)	0	0.243
ERCP‐related complications^b^	4 (6.4)	4 (4.9)	0.726	3 (5.5)	3 (5.5)	1.000

*Note:* Significant *p* values appear in bold.

Abbreviation: ERCP, endoscopic retrograde cholangiopancreatography.

^a^ Categorical variables are presented as *n* (%).

^b^ ERCP‐related complications included bleeding, perforation, respiratory insufficiency, and cardiovascular complications.

### 3.5 Comparative Clinical Outcomes Before and After Matching

Length of hospital and ICU stay did not significantly differ between the groups before or after matching (post‐matching *p* = 0.201 and *p* = 0.606, respectively). There were no significant differences between the groups in terms of prolonged hospital stay and ICU admission (post‐matching *p* = 0.506 and *p* = 0.680, respectively). In‐hospital mortality rates were comparable between the groups before and after matching (*p* = 0.758 and *p* = 0.161, respectively). Before matching, local complications of pancreatitis were more frequent in the non‐urgent ERCP group (14.5% vs. 29.2%, *p* = 0.037), but this difference was not observed post‐matching (*p* = 0.449). There were no significant differences between the groups in terms of pancreatitis severity and systemic complications both before and after matching (post‐matching *p* = 0.326 and *p* = 0.067, respectively).

There were no significant differences between the groups in terms of the composite endpoint, including in‐hospital mortality, prolonged hospital stay, severe pancreatitis, or the development of late localized or systemic complications of pancreatitis, both before and after matching (*p* = 0.751 and *p* = 0.268, respectively). A detailed comparison of clinical outcomes before and after matching is presented in Table 4.

**TABLE 4:** Comparison of clinical outcomes.

Parameters^a^	Entire cohort (before matching)			Matched cohort (after matching)		
	ERCP ≤ 24 h *n* = 62	ERCP 24–72 h *n* = 82	*p*	ERCP ≤ 24 h *n* = 55	ERCP 24–72 h *n* = 55	*p*
Length of hospital stay, day	9 (6–15)	9 (5–12)	0.556	8 (6–15)	10 (6–16)	0.201
Prolonged hospital stay	16 (25.8)	19 (23.2)	0.715	12 (21.8)	15 (27.3)	0.506
ICU admission	21 (33.9)	20 (24.4)	0.212	16 (29.1)	18 (32.7)	0.680
Length of ICU stay, day	8 (5–13)	8 (5–16)	0.685	7 (5–12)	9 (4–16)	0.606
Inotrope requirement	5 (8.1)	8 (9.8)	0.726	3 (5.5)	8 (14.5)	0.112
In‐hospital mortality	4 (6.5)	7 (8.5)	0.758	2 (3.6)	7 (12.7)	0.161
Bacteremia	17 (27.4)	17 (20.7)	0.349	13 (23.6)	15 (27.3)	0.662
Gram‐negative	13 (21)	12 (14.6)		10 (18.2)	10 (18.2)	
Gram‐positive	4 (6.5)	5 (6.1)		3 (5.5)	5 (9.1)	
Pancreatitis severity			0.572			0.326
Mild	43 (69.4)	51 (62.2)		40 (72.7)	37 (67.3)	
Moderate	12 (19.4)	22 (26.8)		11 (20)	9 (16.4)	
Severe	7 (11.3)	9 (11)		4 (7.3)	9 (16.4)	
Necrosis development	2 (3.2)	2 (2.4)	1.000	2 (3.6)	2 (3.6)	1.000
Infected necrosis	1 (1.6)	1 (1.2)	1.000	1 (1.8)	1 (1.8)	1.000
Local complications	9 (14.5)	24 (29.2)	**0.037**	8 (14.5)	11 (20)	0.449
Acute peripancreatic fluid collection	7 (11.3)	20 (24.4)		6 (10.9)	8 (14.5)	
Acute necrotic collection	0	1 (1.2)		0	1 (1.8)	
Walled‐off necrosis	2 (3.2)	1 (1.2)		2 (3.6)	1 (1.8)	
Pseudocyst	0	2 (2.4)		0	1 (1.8)	
Splanchnic venous thrombosis	1 (1.6)	1 (1.2)		1 (1.8)	1 (1.8)	
Systemic complications	5 (8.1)	9 (11)	0.559	3 (5.5)	9 (16.4)	0.067
Composite outcome^b^	15 (24.2)	18 (21.9)	0.751	11 (20)	16 (29.1)	0.268

*Note:* Significant *p* values appear in bold.

Abbreviations: ERCP, endoscopic retrograde cholangiopancreatography; ICU, intensive care unit.

^a^ Categorical variables are presented as *n* (%), non‐normally distributed numerical variables as median (first quartile, third quartile).

^b^ Include mortality, prolonged hospital stay, severe pancreatitis, and late localized or systemic complications of pancreatitis.

Subgroup analyses are summarized in Tables S1–S7. In the non‐severe cholangitis subgroup (Grade I/II), the composite outcome was not significantly different between groups (*p* = 1.000) (Table S1). Similarly, in the severe cholangitis subgroup (Grade III), although the composite outcome favored the urgent ERCP group, the difference did not reach statistical significance (*p* = 0.119) (Table S2). Additionally, clinical and laboratory parameters at admission and prior to ERCP were compared in the non‐urgent group, revealing no significant changes in cholangitis severity, total bilirubin, or procalcitonin levels, while CRP levels increased and several other laboratory markers improved before the procedure (Table S3).

### 3.6 Analysis of Patients Who Did Not Undergo ERCP Within the First 72 h

In our study, we also compared patients who did not undergo ERCP within the first 72 h with those who underwent ERCP within the first 72 h in a population of 327 patients diagnosed with AC and concurrent AGP. Of the 183 patients who did not undergo ERCP within the first 72 h, 25 underwent ERCP between 72 and 120 h, 34 after 120 h, and 124 did not undergo ERCP at all. On admission radiologic imaging, biliary dilation was observed in 147 patients (80.3%), while stones or sludge were detected in 36 patients (19.7%). MRCP was performed in 154 (84.1%) patients after hospitalization. Biliary dilation was not detected in 92 (50.3%) patients, while 16 (8.7%) patients had biliary dilation, and 36 (19.7%) patients had stones or sludge (Table S4). When comparing the general demographic and clinical characteristics between the group that underwent ERCP within 72 h and the conservative treatment group, stones and sludge were more frequently observed in initial radiological imaging in the early ERCP group, whereas biliary dilation was more common in the conservative treatment group (*p* < 0.001) (Table S5).

Cholangitis severity, CRP, and procalcitonin levels were significantly higher in the ERCP within 72 h group (*p* < 0.001) (Table S6). Pancreatitis local complications were more frequently observed in the conservative treatment group (*p* = 0.002). Additionally, the severity of pancreatitis was also higher in the conservative treatment group (*p* = 0.028). There was no significant difference between the groups in terms of the composite endpoint (*p* = 0.950) (Table S7).

## 4 Discussion

In this retrospective study, we investigated the impact of urgent ERCP on clinical outcomes in patients with AC concomitant with AGP. To minimize the effect of confounding factors and reduce selection bias, we performed PSM analysis. Comparison of the matched groups revealed that urgent ERCP did not improve the composite outcome, which included in‐hospital mortality, prolonged hospital stay, severe pancreatitis, and late localized or systemic pancreatic complications, nor did it improve secondary outcomes. Based on our knowledge and literature review, this is the first study to evaluate the impact of urgent ERCP on clinical outcomes in patients with AC concomitant with AGP.

In addition to antibiotic therapy and intravenous fluid resuscitation, timely ERCP is the cornerstone of AC treatment [21]. The role of early ERCP in AC has been a topic of investigation for years. A large study demonstrated that urgent ERCP within 24 h reduced the length of hospital stay but had no effect on mortality [22]. Similarly, other studies have reported that urgent ERCP does not impact mortality [23, 24]. Conversely, some studies suggest that urgent ERCP improves clinical outcomes, including mortality, particularly in patients with severe AC [25, 26]. Unlike urgent ERCP, early ERCP performed within the first 48 h has a clearer impact on prognosis. Multiple studies have shown that early ERCP within 48 h improves prognosis [25, 27, 28]. The TG18, the primary reference for AC management, also recommends urgent ERCP for severe AC [5, 21].

While there is extensive knowledge on ERCP timing in AC, data on ERCP in AGP are more limited. Some studies report that early ERCP improves AGP prognosis, while others have found no significant impact on clinical outcomes [7, 8, 9]. Current acute pancreatitis guidelines do not provide clear recommendations on urgent ERCP, although they state that early ERCP may be performed in cases with concomitant AC [10, 11]. The presence of AGP alongside AC can influence various aspects from diagnosis to treatment and prognosis. As AGP is an inflammatory condition, it leads to elevated CRP levels [29]. Patients with choledocholithiasis presenting with AGP may be diagnosed with AC if they have biliary dilatation and elevated CRP levels, which may cause uncertainty in ERCP timing for clinicians. This highlights a potential gap in TG18, which serves as the primary diagnostic reference for AC. In our study, TG18 criteria were applied strictly to diagnose AC, and only patients who underwent ERCP within the first 72 h were included to ensure that those classified as AC were indeed considered AC cases by clinicians.

Determining the optimal timing of ERCP in patients with both AC and AGP remains a key clinical challenge. In practice, ERCP timing is usually guided by AC severity. However, AGP introduces several complexities. Both AC and AGP share a common etiology (gallstones and biliary sludge) which can spontaneously pass [30]. Stones located more distally in the biliary tract are more likely to drain spontaneously, and several studies have shown a higher rate of spontaneous drainage in patients with concurrent AC and AGP [31]. Moreover, spontaneous drainage is more frequent in patients with AC concomitant with AGP [13]. This is a critical consideration when evaluating the necessity and timing of urgent ERCP.

Another key issue is that pancreatitis is an inflammatory condition, and clinical improvement is typically expected after initial fluid resuscitation. Given that ERCP carries its own risks, including pancreatitis, bleeding, or infection, clinicians may prefer to delay the procedure until the initial inflammatory phase has subsided. To explore these issues, we compared patients who underwent ERCP within 24 h versus those who had the procedure between 24 and 72 h. This question is particularly difficult to answer due to the influence of disease severity on clinical decision‐making. Since urgent ERCP is typically performed in more severe cases, there is inherent selection bias. To minimize this effect, our PSM model controlled for the severity of both AC and AGP, along with other relevant clinical parameters.

Although urgent ERCP appeared to be associated with a trend toward improved clinical outcomes, the difference between urgent and non‐urgent ERCP did not reach statistical significance in patients with AC and concomitant AGP. In the subgroup analysis of patients with severe AC (Grade III), systemic complications and in‐hospital mortality showed a greater tendency toward significance. This limited number of patients in the subgroup analysis may have reduced the statistical power and made interpretation more difficult. However, even in the absence of statistical significance, a favorable trend in clinical outcomes was observed in the urgent ERCP group among severe cases. These findings may reflect the clinical complexity and dynamic course of patients with concomitant AC and AGP, where the decision to perform urgent ERCP must balance potential benefits with procedural risks. The observed favorable trend in the severe subgroup aligns with existing recommendations in the TG18, which advocate for urgent intervention in severe AC. However, the absence of statistical significance, despite this trend, underscores the need for individualized risk–benefit assessment rather than a one‐size‐fits‐all approach. This nuance becomes especially important in settings where diagnostic uncertainty exists due to overlapping inflammatory markers from AGP, or where resource limitations necessitate prioritization of endoscopic procedures.

This study has several limitations. First, its retrospective design carries an inherent risk of selection bias and confounding, despite efforts to minimize these through strict inclusion criteria and PSM. Second, although we used the TG18 for a standardized diagnosis of AC, some patients may have met the criteria due to inflammatory markers elevated by AGP alone. To mitigate this, we included only patients who underwent ERCP within the first 72 h, ensuring consistency in clinical decision‐making. Third, although all eligible patients within the study period were included, the relatively small sample size (particularly in subgroup analyses) may have limited statistical power. This is especially relevant for patients with severe AC, where reduced sample size may have made it more difficult to detect significant differences despite favorable trends. Nevertheless, the main findings of the study remain robust and clinically valuable, offering important insights into a patient population that is underrepresented in the current literature. Finally, variations in ERCP techniques and endoscopist experience could not be fully standardized or accounted for, potentially introducing a source of bias that is inherent to retrospective study designs.

In conclusion, our study provides important insights into ERCP timing in patients with concomitant AGP and AC. Although urgent ERCP did not significantly improve overall clinical outcomes, a favorable trend was observed, particularly in patients with severe AC. These findings suggest that ERCP may not need to be performed urgently in all cases and can potentially be delayed up to 72 h in selected patients without adverse impact on outcomes. However, earlier intervention may still be necessary in severe cases. As a pilot study, our findings underscore the need for larger, multicenter prospective studies to develop evidence‐based strategies for managing this complex patient population.

## Conflicts of Interest

The authors declare no conflicts of interest.

## Supporting information


Table S1.

## Data Availability

The data that support the findings of this study are available on request from the corresponding author. The data are not publicly available due to privacy or ethical restrictions.
