# Supplementary material for: Effect of Urgent ERCP on Clinical Outcomes in Acute Cholangitis With Concurrent Acute Gallstone Pancreatitis: A Propensity Score Matching Analysis
Source: J Hepatobiliary Pancreat Sci. 2025 Jun 5;32(8):591–601. doi: 10.1002/jhbp.12164 (PMC12380036; doi:10.1002/jhbp.12164)
Supplement: Supplementary file 1 — Table S1. [file JHBP-32-591-s001.docx]

**SUPPLEMENTARY TABLES**

| **Table S1.** Comparison of clinical outcomes in the non-severe cholangitis (Grade I/II) subgroup (n=77) | | | |
| --- | --- | --- | --- |
| **Parameters^*^** | **ERCP ≤ 24h**  **n = 39** | **ERCP 24-72h**  **n = 38** | **p** |
| Length of hospital stay, day | 8 (5-13) | 9 (6-11) | 0.334 |
| Prolonged hospitalization | 3 (7.7) | 3 (7.9) | 1.000 |
| ICU admission | 5 (12.8) | 5 (13.2) | 1.000 |
| Length of ICU stay, day | 6 (3-12) | 14 (4-22) | 0.341 |
| Inotrope requirement | 1 (2.6) | 1 (2.6) | 1.000 |
| In-hospital mortality | 1 (2.6) | 1 (2.6) | 1.000 |
| *Bacteremia* |  |  | 0.591 |
| Gram-negative | 8 (20.5) | 5 (13.2) |  |
| Gram-positive | 0 | 1 (2.6) |  |
| *Pancreatitis severity^**^* |  |  | 0.494 |
| Mild | 32 (82.1) | 33 (86.8) |  |
| Moderate | 7 (17.9) | 4 (10.5) |  |
| Severe | 0 | 1 (2.6) |  |
| Necrosis development | 1 (2.6) | 2 (5.3) | 0.615 |
| Infected necrosis | 0 | 1 (2.6) | 0.494 |
| *Local complications* | 6 (15.4) | 5 (13.2) | 0.780 |
| Acute peripancreatic fluid collection | 5 (12.8) | 2 (5.3) |  |
| Acute necrotic collection | 0 | 1 (2.6) |  |
| Walled-off necrosis | 1 (2.6) | 1 (2.6) |  |
| Pseudocyst | 0 | 1 (2.6) |  |
| Splanchnic venous thrombosis | 0 | 1 (2.6) |  |
| Systemic complications | 1 (2.6) | 1 (2.6) | 1.000 |
| Composite outcome^***^ | 4 (10.3) | 4 (10.5) | 1.000 |
| *^*^Categorical variables are presented as n (%), non-normally distributed numerical variables as median (first quartile, third quartile), **Comparison was made between severe and non-severe groups ^***^Include mortality, prolonged hospital stay, severe pancreatitis, and late localized or systemic complications of pancreatitis.* | | | |
|  | | | |

| **Table S2.** Comparison of clinical outcomes in the severe cholangitis (Grade III) subgroup (n=33) | | | |
| --- | --- | --- | --- |
| **Parameters^*^** | **ERCP ≤ 24h**  **n = 16** | **ERCP 24-72h**  **n = 17** | **p** |
| Length of hospital stay, day | 13 (7-20) | 17 (8-29) | 0.226 |
| Prolonged hospitalization | 6 (37.5) | 9 (52.9) | 0.373 |
| ICU admission | 11 (68.8) | 13 (76.5) | 0.708 |
| Length of ICU stay, day | 7 (5-11) | 6 (5-16) | 0.884 |
| Inotrope requirement | 2 (12.5) | 7 (41.2) | 0.118 |
| In-hospital mortality | 1 (6.3) | 6 (35.3) | 0.085 |
| *Bacteremia* |  |  | 0.208 |
| Gram-negative | 2 (12.5) | 5 (29.4) |  |
| Gram-positive | 3 (18.8) | 4 (23.5) |  |
| *Pancreatitis severity^**^* |  |  | 0.188 |
| Mild | 8 (50) | 4 (23.5) |  |
| Moderate | 4 (25) | 5 (29.4) |  |
| Severe | 4 (25) | 8 (47.1) |  |
| Necrosis development | 1 (6.3) | 0 | 0.485 |
| Infected necrosis | 1 (6.3) | 0 | 0.485 |
| *Local complications* | 2 (12.5) | 6 (35.3) | 0.225 |
| Acute peripancreatic fluid collection | 1 (6.3) | 6 (35.3) |  |
| Acute necrotic collection | 0 | 0 |  |
| Walled-off necrosis | 1 (6.3) | 0 |  |
| Pseudocyst | 0 | 0 |  |
| Splanchnic venous thrombosis | 1 (6.3) | 0 |  |
| Systemic complications | 2 (12.5) | 8 (47.1) | 0.057 |
| Composite outcome^***^ | 7 (43.8) | 12 (70.6) | 0.119 |
| *^*^Categorical variables are presented as n (%), non-normally distributed numerical variables as median (first quartile, third quartile), **Comparison was made between severe and non-severe groups ^***^Include mortality, prolonged hospital stay, severe pancreatitis, and late localized or systemic complications of pancreatitis.* | | | |

| **Table S3**. Comparison of clinical parameters at admission and before ERCP in patients undergoing non-urgent ERCP (n=55) | | | |
| --- | --- | --- | --- |
| **Parameters** | **At Admission** | **Pre-ERCP** | **p** |
| *Cholangitis severity* |  |  | 0.223 |
| Grade 1 (mild) | 23 (41.8) | 28 (50.9) |  |
| Grade 2 (moderate) | 15 (27.3) | 11 (20) |  |
| Grade 3 (severe) | 17 (30.9) | 16 (29.1) |  |
| White blood cell count, 10^9^/L | 12 (9-15) | 10.8 (6.7-14.1) | 0.015 |
| Hemoglobin, g/dL | 13.6 ± 2 | 12.7 ± 2.2 | <0.001 |
| Platelet count, 10^9^/L | 230 (186-293) | 216 (152-270) | 0.017 |
| Urea, mg/dL | 39 (28-58) | 40 (26-60) | 0.362 |
| Creatinine, mg/dL | 0.9 (0.7-1.2) | 0.78 (0.63-1.04) | 0.118 |
| Albumin, g/dL | 4 (3.6-4.4) | 3.5 (3.3-4.1) | <0.001 |
| Aspartate Aminotransferase, U/L | 177 (94-355) | 108 (58-222) | <0.001 |
| Alanine Aminotransferase, U/L | 210 (112-422) | 170 (93-281) | <0.001 |
| Alkaline Phosphatase, U/L | 259 (173-424) | 234 (142-383) | <0.001 |
| Gamma-Glutamyl Transferase, U/L | 422 (280-583) | 343 (200-499) | <0.001 |
| Lactate Dehydrogenase, U/L | 351 (291-480) | 286 (210-411) | <0.001 |
| Total bilirubin, mg/dL | 5.3 (3-7) | 4.68 (2.38-7.44) | 0.527 |
| Amylase, U/L | 880 (458-1843) | 277 (121-899) | <0.001 |
| Lipase, U/L | 1270 (540-2911) | 266 (93-717) | <0.001 |
| C-reactive protein, mg/L | 54 (24-152) | 121 (52-170) | <0.001 |
| Procalcitonin, µg/L | 0.86 (0.2-14.4) | 0.88 (0.21-11.82) | 0.928 |
| *^*^Categorical variables are presented as n (%), non-normally distributed numerical variables as median (first quartile, third quartile), and normally distributed numerical variables as mean ± standard deviation.* | | | |

| **Table S4.** Clinical and radiological characteristics of the group without ERCP within the first 72 hours | |
| --- | --- |
| **Parameters** | **n = 183** |
| *ERCP time, hours, n (%)* |  |
| 72–120 hours | 25 (13.6) |
| After 120 hours | 34 (18.6) |
| Not performed | 124 (67.8) |
| Gallbladder stones or sludge, n (%) | 136 (74.3) |
| *Imaging finding on admission, n (%)* |  |
| Biliary dilatation | 147 (80.3) |
| Stone or sludge | 36 (19.7) |
| Advanced imaging after 48 hours, n (%) | 144 (78.7) |
| *ERCP finding, n (%)* |  |
| Presence of stones | 38 (20.8) |
| Presence of sludge/debris | 9 (4.9) |
| Clear biliary ducts | 12 (6.6) |
| *MRCP finding, n (%)* |  |
| No biliary dilatation | 92 (50.3) |
| Biliary dilatation | 16 (8.7) |
| Stones or sludge | 36 (19.7) |
| Not performed | 29 (15.8) |
| *Abbreviations: ERCP; endoscopic retrograde cholangiopancreatography, MRCP; magnetic resonance cholangiopancreatography.* | |

| **Table S5.** Comparison of general demographic and clinical characteristics of the entire study population (n=327) | | | |
| --- | --- | --- | --- |
| **Parameters^*^** | **ERCP ≤72h**  **n = 144** | **Conservative treatment**  **n = 183** | **p** |
| Age, years | 66 ± 16 | 63 ± 18 | 0.236 |
| Female gender | 68 (47.2) | 104 (56.8) | 0.084 |
| *Comorbidities* |  |  |  |
| Diabetes mellitus | 43 (29.9) | 41 (22.4) | 0.125 |
| Hypertension | 65 (45.1) | 96 (52.5) | 0.189 |
| Cardiovascular disease | 33 (22.9) | 46 (25.1) | 0.642 |
| Cerebrovascular disease | 6 (4.2) | 12 (6.6) | 0.347 |
| CCI score | 1 (0-3) | 1 (0-2) | 0.688 |
| Abdominal pain | 143 (99.3) | 179 (97.8) | 0.389 |
| Jaundice | 65 (45.1) | 54 (29.5) | 0.004 |
| *Vital signs* |  |  |  |
| Mean arterial pressure | 88 (77-93) | 88 (80-97) | 0.137 |
| Heart rate per minute | 84 (78-98) | 82 (76-90) | 0.140 |
| Oxygen saturation, % | 95 (93-97) | 95 (93-96) | 0.739 |
| Glasgow Coma score <15 | 10 (6.9) | 7 (3.8) | 0.207 |
| Recurrent pancreatitis | 13 (9) | 30 (16.4) | 0.050 |
| Concomitant cholecystitis | 27 (18.8) | 34 (18.6) | 0.969 |
| History of cholecystectomy | 27 (18.8) | 25 (13.7) | 0.212 |
| *Imaging finding on admission* |  |  | **<0.001** |
| Biliary dilatation | 62 (43.1) | 147 (80.3) |  |
| Stone or sludge | 82 (56.9) | 36 (19.7) |  |
| Advanced imaging after 48h | 36 (25) | 144 (78.7) | **<0.001** |
| *MRCP finding* |  |  | **<0.001** |
| Biliary dilatation | 6 (4.2) | 16 (8.7) |  |
| No biliary dilatation | 5 (3.5) | 92 (50.3) |  |
| Stones or sludge | 1 (0.7) | 36 (19.7) |  |
| Not performed | 132 (91.7) | 29 (15.8) |  |
| Gallbladder stones or sludge | 100 (69.4) | 136 (74.3) | 0.329 |
| *^*^Categorical variables are presented as n (%), non-normally distributed numerical variables as median (first quartile, third quartile), and normally distributed numerical variables as mean ± standard deviation. Abbreviations: ERCP; endoscopic retrograde cholangiopancreatography, CCI; Charlson comorbidity index, MRCP; magnetic resonance cholangiopancreatography.* | | | |

| **Table S6.** Comparison of classifications of disease severity and laboratory parameters of the entire study population (n=327) | | | |
| --- | --- | --- | --- |
| **Parameters^*^** | **ERCP ≤72h**  **n = 144** | **Conservative treatment**  **n = 183** | **p** |
| ERCP time, hours | 33 (19-44) | 133 (116-200) | **<0.001** |
| *ERCP finding* |  |  | **0.010** |
| Presence of stones | 104 (72.2) | 38 (20.8) |  |
| Presence of sludge/debris | 31 (21.5) | 9 (4.9) |  |
| Clear biliary ducts | 9 (6.3) | 12 (6.6) |  |
| ERCP-related complications | 8 (5.5) | 8 (4.4) | 0.622 |
| *Cholangitis severity* |  |  | **<0.001** |
| Grade 1 (mild) | 56 (38.9) | 118 (64.5) |  |
| Grade 2 (moderate) | 49 (34) | 41 (22.4) |  |
| Grade 3 (severe) | 39 (27.1) | 24 (13.1) |  |
| qSOFA score ≥ 2 | 10 (6.9) | 4 (2.2) | **0.035** |
| SIRS score ≥ 2 | 41 (28.5) | 40 (21.9) | 0.169 |
| *Scoring systems* |  |  |  |
| Ranson | 3 (2-4) | 3 (1-4) | 0.192 |
| BISAP | 1 (0-2) | 1 (0-2) | 0.253 |
| APACHE II | 7 (3-10) | 6 (4-8) | 0.469 |
| Glasgow-Imrie | 1 (1-2) | 1 (1-2) | 0.103 |
| Organ failure at admission | 17 (11.8) | 21 (11.5) | 0.926 |
| CRP >150 mg/dL, first 48 hours | 60 (41.7) | 58 (31.7) | 0.062 |
| Pleural effusion, first 48 hours | 12 (8.3) | 21 (11.5) | 0.349 |
| *Laboratory parameters* |  |  |  |
| White blood cell count, | 12.1 (9.2-16) | 11.2 (8.3-14.2) | **0.016** |
| Hemoglobin, g/dL | 13.7 ± 2 | 13.7 ± 2 | 0.930 |
| Platelet count, 10^9^/L | 228 (184-284) | 238 (193-287) | 0.261 |
| Creatinine, mg/dL | 1 (0.8-1.3) | 0.9 (0.7-1.1) | **0.004** |
| Albumin, g/dL | 3.9 (3.6-4.3) | 4.2 (3.9-4.4) | **<0.001** |
| Total bilirubin, mg/dL | 5.4 (3-7.4) | 3.1 (1.7-4.4) | **<0.001** |
| Procalcitonin | 1.97 (0.22-10.76) | 0.22 (0.09-2.64) | **<0.001** |
| CRP, mg/L | 62 (23-144) | 23 (10-62) | **<0.001** |
| *^*^Categorical variables are presented as n (%), non-normally distributed numerical variables as median (first quartile, third quartile), and normally distributed numerical variables as mean ± standard deviation.* *^**^ERCP-related complications included bleeding, perforation, respiratory insufficiency, and cardiovascular complications. Abbreviations: ERCP; endoscopic retrograde cholangiopancreatography, qSOFA; quick sequential organ failure assessment, SIRS; systemic inflammatory response syndrome, CRP; C-reactive protein.* | | | |

| **Table S7.** Comparison of clinical outcomes of the entire study population (n=327) | | | |
| --- | --- | --- | --- |
| **Parameters^*^** | **ERCP ≤72h**  **n = 144** | **Conservative treatment**  **n = 183** | **p** |
| Length of hospital stay, day | 9 (6-14) | 9 (7-12) | 0.607 |
| Prolonged hospitalization | 38 (26.4) | 39 (21.3) | 0.283 |
| ICU admission | 41 (28.5) | 49 (26.8) | 0.733 |
| Length of ICU stay, day | 8 (5-14) | 6 (3-12) | 0.184 |
| Inotrope requirement | 13 (9) | 10 (5.5) | 0.211 |
| In-hospital mortality | 11 (7.6) | 8 (4.4) | 0.210 |
| *Bacteremia* | 34 (23.6) | 16 (8.7) | **<0.001** |
| Gram-negative | 25 (17.4) | 12 (6.6) |  |
| Gram-positive | 9 (6.3) | 4 (2.2) |  |
| *Pancreatitis severity* |  |  | **0.028** |
| Mild | 94 (65.3) | 101 (55.2) |  |
| Moderate | 34 (23.6) | 68 (37.2) |  |
| Severe | 16 (11.1) | 14 (7.7) |  |
| Necrosis development | 4 (2.8) | 15 (8.2) | **0.038** |
| Infected necrosis | 2 (1.4) | 2 (1.1) | 1.000 |
| *Local complications* | 33 (22.9) | 71 (38.8) | **0.002** |
| Acute peripancreatic fluid collection | 27 (18.8) | 51 (27.9) |  |
| Acute necrotic collection | 1 (0.7) | 6 (3.4) |  |
| Walled-off necrosis | 3 (2.1) | 9 (4.9) |  |
| Pseudocyst | 2 (1.4) | 4 (2.2) |  |
| Splanchnic venous thrombosis | 2 (1.4) | 3 (1.6) |  |
| Systemic complications | 14 (9.7) | 11 (6) | 0.210 |
| Composite outcome^**^ | 39 (27.1) | 49 (26.8) | 0.950 |
| *^*^Categorical variables are presented as n (%), non-normally distributed numerical variables as median (first quartile, third quartile), ^**^Include mortality, prolonged hospital stay, severe pancreatitis, and late localized or systemic complications of pancreatitis. Abbreviations: ERCP; endoscopic retrograde cholangiopancreatography, ICU; intensive care unit.* | | | |
|  |  |  |  |
